# Efficacy and safety of PD-1/PD-L1 inhibitors as first-line treatment for esophageal squamous cell carcinoma: a systematic review and meta-analysis

**DOI:** 10.3389/fimmu.2025.1563300

**Published:** 2025-03-26

**Authors:** Wei Ren, Hanyu Zhang, Yixin Li, Wu Sun, Hexiang Peng, Huangda Guo, Tianjiao Hou, Mengying Wang, Zhendong Hu, Tao Wu, Baorui Liu

**Affiliations:** ^1^ The Comprehensive Cancer Center of Drum Tower Hospital, Medical School of Nanjing University and Clinical Cancer Institute of Nanjing University, Nanjing, China; ^2^ Department of Epidemiology and Biostatistics, School of Public Health, Peking University, Beijing, China; ^3^ Key Laboratory of Epidemiology of Major Diseases (Peking University), Ministry of Education, Beijing, China; ^4^ Department of Nutrition and Food Hygiene, School of Public Health, Peking University, Beijing, China; ^5^ Department of Esophageal Surgery, Drum Tower Hospital, Medical School of Nanjing University, Nanjing, China

**Keywords:** PD-1/PD-L1 inhibitor, esophageal squamous cell carcinoma, meta-analysis, immunotherapy, combined positive score

## Abstract

**Purpose:**

This study aims to investigate the efficacy and safety of PD-1/PD-L1 inhibitors in the first-line treatment of esophageal squamous cell carcinoma (ESCC) and identify factors influencing efficacy through a meta-analysis of multiple phase 3 randomized controlled trials (RCTs).

**Methods:**

A systematic literature search was conducted in Cochrane, PubMed, and Embase databases. Two researchers independently extracted trial data, including efficacy-related outcomes such as overall survival (OS), progression-free survival (PFS), objective response rate (ORR), and duration of response (DoR), along with their subgroup data and safety-related indicators. The overall hazard ratio (HR) and 95% confidence interval (CI) were calculated for OS and PFS, while the overall odds ratio (OR) and 95% CI were computed for ORR to compare the classification and predictive abilities of combined positive score (CPS) and tumor proportion score (TPS) for PD-L1 status. Additionally, survival outcomes across different subgroups were evaluated to explore the potential influencing factors for the efficacy of PD-1/PD-L1 inhibitors in ESCC.

**Results:**

This meta-analysis included eight phase 3 RCTs encompassing 4,479 participants. PD-1/PD-L1 inhibitors combined with chemotherapy significantly improved OS (*HR*: 0.68, 95% *CI*: 0.63-0.74) and PFS (*HR*: 0.62, 95% *CI*: 0.58-0.67) in ESCC patients compared to non-combination therapy. Patients with higher PD-L1 expression (CPS>1 or TPS>1) demonstrated superior responses to PD-1/PD-L1 inhibitions, with CPS identified as a stronger predictor of therapeutic benefit, particularly at a threshold of CPS =10. Subgroup analysis revealed that male, Asian, smoking, and liver metastasis patients exhibited a greater trend toward improved disease control with PD-1/PD-L1 inhibitors. However, there was no significant difference in treatment efficacy between immune therapy combined with TP (taxol [paclitaxel] + cisplatin) and FP (5-fluorouracil [5-FU] + cisplatin) regimens (*P_OS_* =0.51, *P_PFS_* =0.11). Finally, PD-1/PD-L1 inhibition was associated with a higher incidence of grade ≥3 adverse events compared to chemotherapy alone (*HR*: 1.21, 95% *CI*: 1.07-1.37).

**Conclusions:**

This study confirms that the combination of PD-1/PD-L1 inhibitors and chemotherapy provides significant clinical benefits in ESCC. CPS =10 serves as a key threshold for predicting treatment response. There is a trend suggesting that male, Asian, smoking, and liver metastasis patients may experience better survival benefits, while no significant difference was observed between TP- and FP-based regimens.

**Systematic Review Registration:**

https://www.crd.york.ac.uk/prospero, identifier CRD42024536221

## 1 Introduction

Esophageal cancer is a highly aggressive malignancy of the digestive system and ranks as the seventh leading cause of cancer-related deaths globally (1). Esophageal squamous cell carcinoma (ESCC), which arises from the squamous epithelium of the esophagus, predominantly affects the upper and middle esophageal segments. Due to its highly invasive nature and often asymptomatic or absence of specific early symptoms, it is frequently diagnosed at an advanced stage, with a poor prognosis and a five-year survival rate of approximately 20% (2, 3). The incidence of ESCC is notably higher in Asia, Africa, and South America compared to Western countries (4). Notably, in high-incidence regions such as China, ESCC accounts for more than 90% of esophageal cancer cases (5–7).

The primary treatment modalities for ESCC include surgery, radiotherapy, and chemotherapy (8). Currently, the predominant treatment approaches involve immune checkpoint inhibitors (ICIs) combined with chemotherapy or ICIs alone. For first-line chemotherapy, there are two main options: TP (taxol [paclitaxel] + cisplatin) and FP (5-fluorouracil [5-FU] + cisplatin) (9, 10). In recent years, ICIs have emerged as a promising therapeutic strategy for esophageal cancer, garnering increasing attention. Previous studies have shown that ICIs enhance anti-tumor immunity by blocking immune checkpoint molecules, thereby restoring the immune system’s ability to recognize and attack tumor cells (11, 12). Among ICIs, programmed death 1 (PD-1) and programmed death ligand 1 (PD-L1) inhibitors are of particular significance. PD-L1, an immune inhibitory molecule expressed on activated T cells, B cells, and natural killer (NK) cells, binds to the PD-1 receptor, suppressing T-cell activation and enabling tumor cells to evade immune surveillance.

Several clinical trials and meta-analyses have demonstrated that combining PD-1 inhibitors with chemotherapy improves overall survival (OS) and progression-free survival (PFS) in patients with locally advanced, metastatic, or recurrent ESCC (13–15). However, the optimal chemotherapy regimen to be used in combination with PD-1 inhibitors remains unclear. The efficacy of PD-1 inhibitors in combination with either the FP or TP chemotherapy regimen has shown variability across different randomized controlled trials (RCTs). A recent meta-analysis (13), which included 10 trials, suggested that for advanced, metastatic, or recurrent ESCC, first-line treatment with ICIs+TP may offer superior outcomes compared to ICIs+FP. The ICIs+TP regimen showed significantly better OS and response rates compared to ICIs+FP. In addition, patients receiving ICIs+FP tend to experience more gastrointestinal toxicities, whereas those treated with ICIs+TP are more prone to hematologic toxicities. In clinical decision-making, both the efficacy and toxicity profiles of ICIs, along with the patient’s overall condition, must be carefully considered.

Additionally, PD-L1 expression levels may be associated with clinical benefits in ESCC patients. Previous studies have found that high PD-L1 expression in non-small cell lung cancer (NSCLC) patients is associated with improved efficacy of PD-1/PD-L1 inhibitors (16). However, there is no clear consensus regarding this relationship in ESCC. Two common immunohistochemical methods for assessing PD-L1 expression are the combined positive score (CPS) and the tumor proportion score (TPS). TPS measures the proportion of tumor cells with PD-L1 expression on their membranes, while CPS accounts for PD-L1 expression on both tumor and immune cells relative to the total number of tumor cells (17, 18). Despite these methods, there is no established consensus on the optimal scoring system or threshold for predicting the efficacy of PD-1/PD-L1 inhibitors in cancer patients (19).

In summary, the current study aims to explore the efficacy and safety of PD-1/PD-L1 inhibitors in the treatment of ESCC and identify factors influencing therapeutic outcomes through a meta-analysis of multiple phase III RCTs.

## 2 Materials and methods

### 2.1 Search strategy

**Figure 1:**
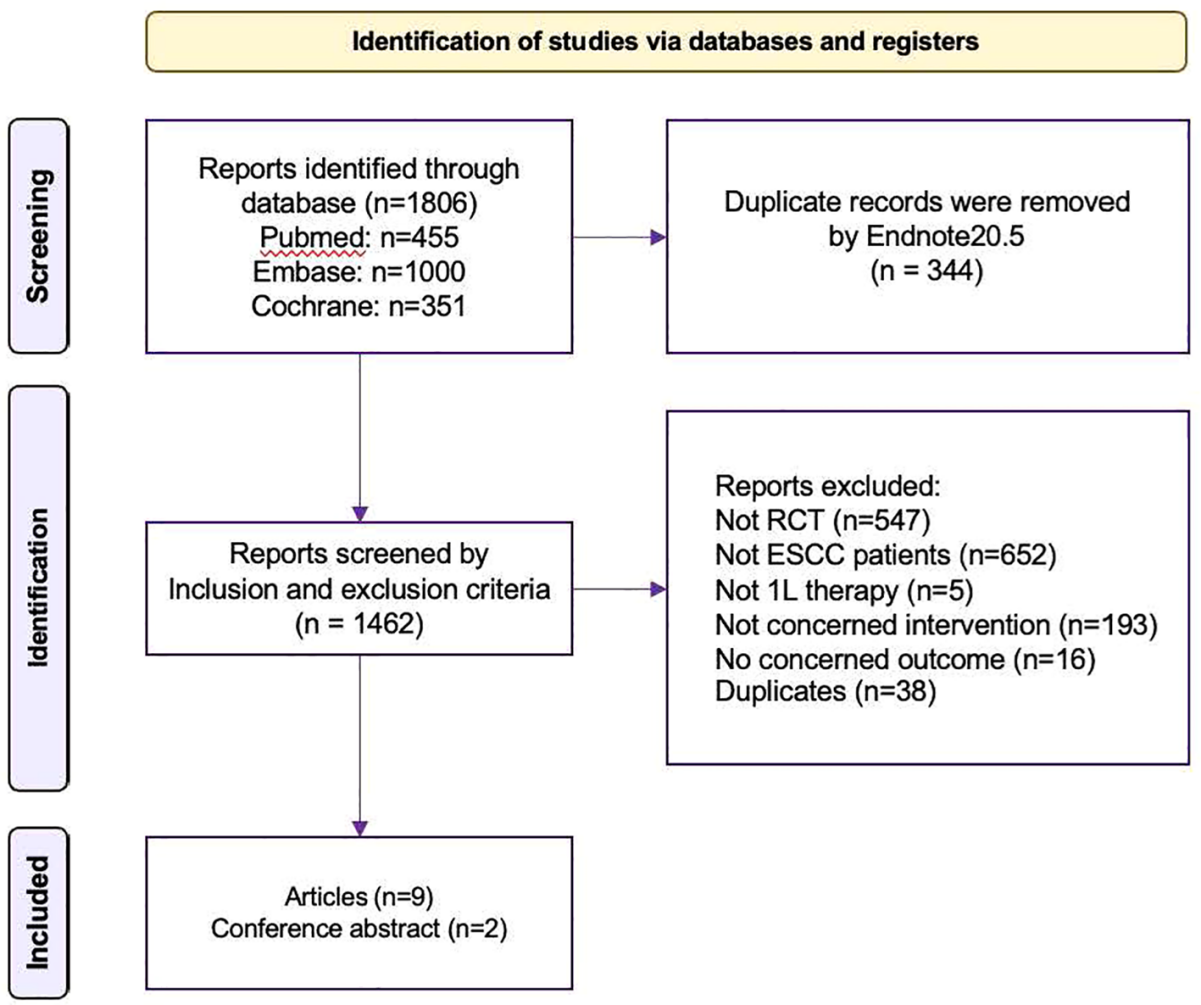
Flow diagram of the identification of eligible studies.

The current study conducted a systematic literature search across multiple databases, including Cochrane (all fields), PubMed, and Embase, covering studies from database inception to July 31, 2024. This study has been registered on the PROSPERO website with registration number CRD42024536221. The search strategy utilized the following keywords: ((esophageal squamous cell carcinoma) OR (esophageal squamous cell cancer) OR (esophageal cancer) OR (esophageal carcinoma)) AND ((serplulimab) OR (sintilimab) OR (Nivolumab) OR (camrelizumab) OR (sugemalimab) OR (Toripalimab) OR (Pembrolizumab) OR (tislelizumab) OR (Immune checkpoint inhibition) OR (PD-1) OR (PD-L1)) AND (placebo OR chemotherapy) AND ((progression-free survival) OR PFS OR (overall survival) OR OS OR (objective response) OR ORR OR (duration of response) OR DoR OR (patient reported outcome) OR PRO OR pain OR (quality of life) OR QoL OR (use of other subsequent therapy*) OR (performance status deterioration) OR (time to clinical progression) OR (time to disease progression) OR (time to pain progression) OR (disease response rate) OR safety OR tolerability OR (adverse outcome*) OR (adverse event*) OR AE) AND (trial* OR random*). A PRISMA flow diagram illustrating the study selection process is presented in **Figure 1**.

### 2.2 Inclusion and exclusion criteria

Inclusion criteria were as follows: (1) The study participants were diagnosed with ESCC. (2) The studies were phase III RCTs of ESCC. (3) Efficacy was evaluated based on PD-L1 metrics, either CPS or TPS. (4) The studies involved first-line treatment regimens. (5) The RCT interventions included PD-1/PD-L1 inhibitors with or without chemotherapy. (6) The studies reported available efficacy outcomes.

Exclusion criteria were as follows: (1) Studies in which the population or interventions did not meet the inclusion criteria. (2) Studies that did not report the outcomes of interest.

### 2.3 Data extraction and quality assessment

Two researchers independently extracted data from each trial, including the following: (1) Study details: clinical trial name, first author, publication year, country, registered NCT number, and RCT phase. (2) Participant information: sample sizes for the control and intervention groups, median/mean age, and follow-up duration. (3) Group information: intervention and drug dosages, control group measures and drug dosages. (4) Efficacy outcomes: OS, PFS, objective response rate (ORR), duration of response (DoR), and subgroup data. (5) Safety outcomes: Incidence rates of any adverse events and serious adverse events.

### 2.4 Statistical analysis

All analyses were conducted using R software (version 4.2.2). First, the overall hazard ratios (HRs) and 95% confidence intervals (CIs) for OS and PFS were calculated, along with overall odds ratios (ORs) and 95% CIs, to evaluate the efficacy and safety of PD-1/PD-L1 inhibitors in ESCC. Next, the influence of different PD-L1 expression levels on survival outcomes was analyzed, comparing the classification and predictive capabilities of CPS and TPS for PD-L1 status. Finally, survival outcomes across different subgroups were assessed to identify potential factors influencing the efficacy of PD-L1 inhibitors in ESCC treatment. The *I²* statistic was used to assess the heterogeneity among studies, with an *I²* value of <50% indicating low heterogeneity, warranting the use of a fixed-effects model, and an *I²* ≥50% indicating high heterogeneity, necessitating a random-effects model. A two-sided p-value of <0.05 was considered statistically significant.

## 3 Results

### 3.1 Study characteristics

A total of 1,806 relevant articles were identified in the literature search. After excluding 344 duplicate records and 1451 articles that did not meet the inclusion criteria, 9 full-text articles and 2 conference abstracts were included in the final analysis (10, 14, 15, 20–27). The nine articles covered eight phase III RCTs, including two subgroup analyses of the KEYNOTE-590 and CheckMate 648 trials in the Japanese population. Relevant baseline characteristics are summarized in **Table 1**. A total of 4,479 patients were included across the 8 RCTs, all of which focused on first-line treatments. Among these trials, three studies compared the treatment efficacy of PD-1/PD-L1 inhibitors combined with FP regimens versus placebo plus FP regimens, while another 3 trials assessed the treatment outcomes of PD-1/PD-L1 inhibitors combined with TP regimens versus placebo plus TP regimens. Additionally, one trial compared the efficacy of PD-1 inhibitors plus chemotherapy against a combination of PD-1 inhibitors and CTLA-4 inhibitors in immunotherapy for ESCC. The ORIENT-15 and RATIONALE-306 trials further compared the efficacy of immunotherapy combined with different chemotherapy regimens.

**Table 1:** Characteristics of the included trials.

Clinical Trials	Group	N	HR for OS (95%CI)	HR for PFS (95%CI)	ORR (%)	DoR (months)
ASTRUM-007	serplulimab + FP	368	0.68(0.53,0.87)	0.6(0.48,0.75)	57.61%	6.9(5.6,8.3)
	placebo + FP	183			42.08%	4.6(4.1,5.6)
CheckMate 648	nivolumab + FP	321	0.74(0.58,0.96)	0.81(0.64,1.04)	47.35%	8.2 (6.9,9.7)
	nivolumab + ipilimumab	325			27.69%	11.1(8.3,14.0)
	FP	324			26.85%	7.1 (5.7,8.2)
ESCORT-1st	camrelizumab + TP	298	0.70(0.56,0.88)	0.56(0.46,0.68)	72.15%	7.0(6.1,8.9)
	placebo + TP	298			62.08%	4.6(4.3,5.5)
GEMSTONE-304	Sugemalimab + TP	358	0.70(0.55,0.90)	0.67(0.54,0.82)	58.38%	6.0(5.5,7.0)
	Placebo + TP	182			43.96%	4.5(4.1,5.3)
JUPITER-06	Toripalimab + TP	257	0.58(0.43,0.78)	0.58(0.46,0.74)	69.26%	5.6(4.48.7)
	Placebo + TP	257			52.14%	4.2(4.2,4.4)
KEYNOTE-590	pembrolizumab + FP	373	0.73(0.61,0.88)	0.65(0.54,0.78)	45.04%	8.3(1.2,31.0)
	Placebo + FP	376			29.26%	6.0(1.5,25.0)
ORIENT-15	sintilimab + chemo(FP or TP)	327	0.63(0.51,0.78)	0.56(0.46,0.68)	66.06%	9.7(7.1,13.7)
	placebo + chemo(FP or TP)	332			45.48%	6.9(5.6,7.2)
RATIONALE-306	Tislelizumab + chemo(FP or TP)	326	0.66(0.54,0.80)	0.62(0.52,0.75)	63.50%	7.1(6.1,8.1)
	Placebo + chemo(FP or TP)	323			42.41%	5.7(4.4,7.1)

### 3.2 Long‐term efficacy outcomes: overall survival and progression‐free survival

**Figure 2:**
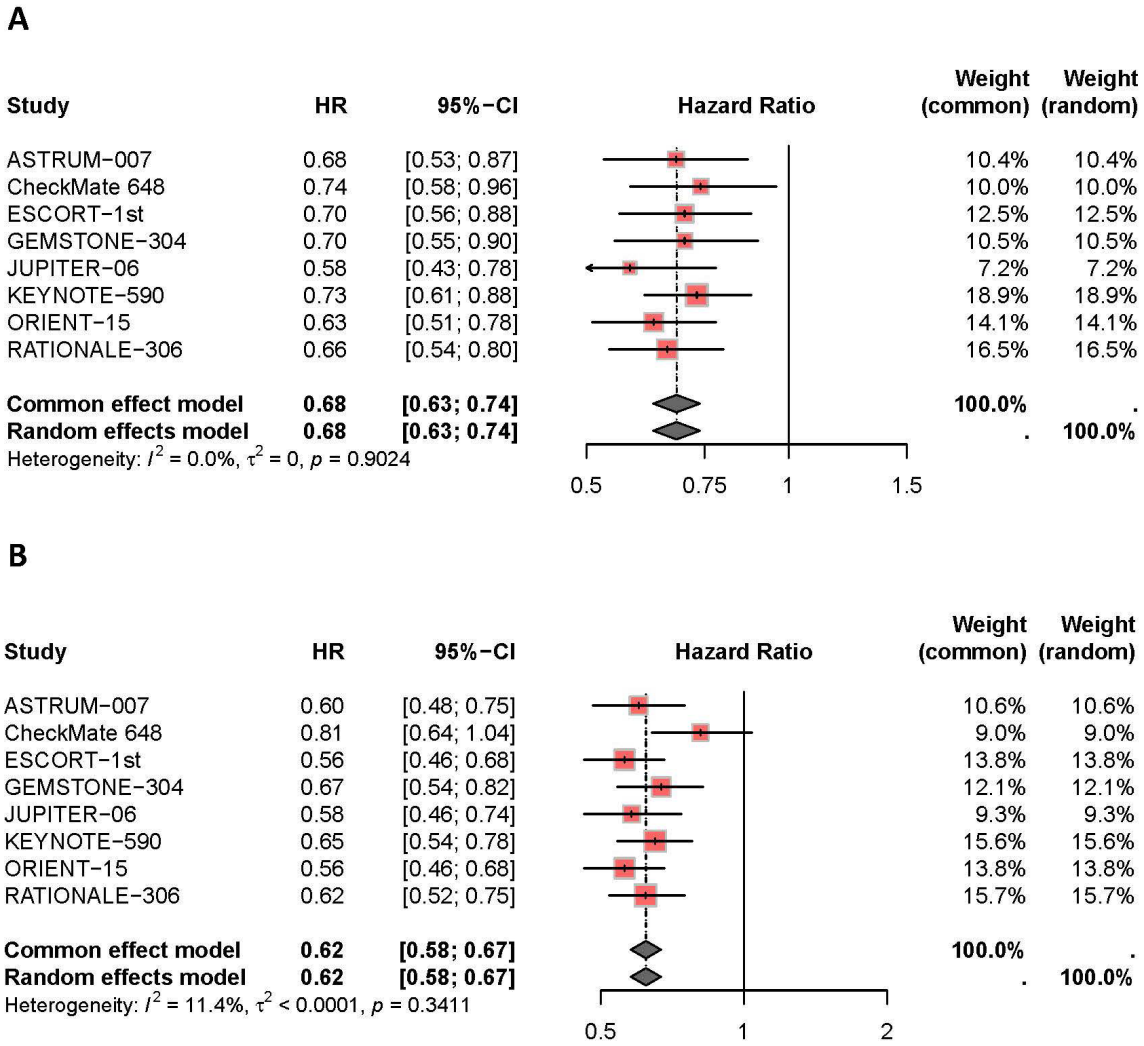
Compared with the control group, the overall Hazard Ration and 95% Confidence Interval (CI) of OS **(A)** and PFS **(B)** in patients treated with a combination therapy of PD-1/PD-L1 inhibitors and chemotherapy.

A meta-analysis was performed to evaluate OS and PFS based on data from the 8 included RCTs, as shown in **Figure 2**. Compared to non-combination therapies, the addition of PD-1/PD-L1 inhibitors to chemotherapy significantly prolonged OS in ESCC patients (*I^2^ =* 0%, HR: 0.68, 95% CI: 0.63-0.74). Similarly, the combination of PD-1/PD-L1 inhibitors with chemotherapy showed a better PFS compared to chemotherapy alone for ESCC patients (*I^2^ =* 11%, HR: 0.62, 95% CI: 0.58-0.67).

### 3.3 PD-L1 status

CPS and TPS are commonly used scoring systems for evaluating PD-L1 expression. The present study analyzed treatment outcomes in patients with varying PD-L1 expression levels, comparing the classification thresholds of CPS and TPS and their correlation with therapy selection and prognosis.

**Figure 3:**
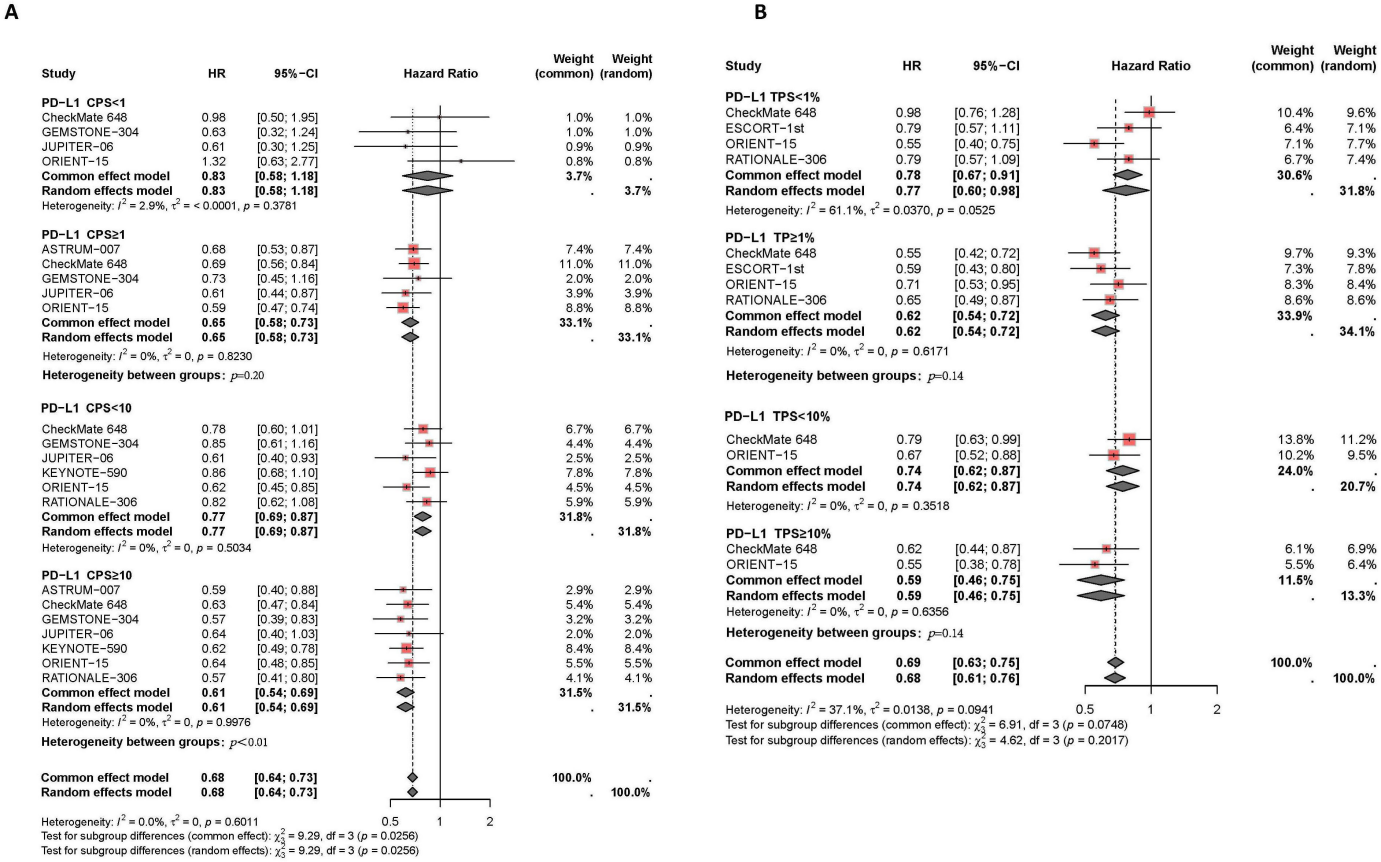
Forest plot of subgroup analysis comparing the overall survival HR in patients who received PD-1/PD-L1 inhibitor-based therapy versus chemotherapy based on different PDL1 expression levels of CPS **(A)** and TPS **(B)**.

Patients with high PD-L1 expression showed a better response to PD-1/PD-L1 inhibitor therapy, with a lower risk of death compared to those with low PD-L1 expression, as illustrated in **Figure 3**. Subgroup analysis based on different CPS thresholds revealed significant differences between groups (*P*=0.03). In patients with CPS <1, there was no significant difference in the risk of death between those receiving PD-1/PD-L1 inhibitors combined with chemotherapy and those receiving chemotherapy alone (*I^2^ =* 3%, HR: 0.83, 95% CI: 0.58-1.18). However, in patients with CPS ≥1, the combination of PD-1/PD-L1 inhibitors with chemotherapy reduced the risk of death compared to the control group (*I^2^ =* 0%, HR: 0.65, 95% CI: 0.58-0.73). For patients with CPS ≥10, the reduction in death risk with the combination therapy was even more pronounced compared to those with CPS <10 (*I^2^ =* 0%, HR: 0.61, 95% CI: 0.54-0.69, versus I²=0%, HR: 0.77, 95% CI: 0.69-0.87, *P*<0.01). Subgroup analysis based on TPS thresholds did not yield significant differences between groups (*P*=0.07). Compared to patients with TPS <1% (*I^2^ =* 61%, HR: 0.77, 95% CI: 0.60-0.98), those with TPS ≥1% experienced a reduction in the risk of death with the combination therapy compared to the control group (*I^2^ =* 0%, HR: 0.62, 95% CI: 0.54-0.72). Similarly, in patients with TPS ≥10%, the reduction in death risk with the combination therapy was slightly more pronounced than in those with TPS <10% (*I^2^ =* 0%, HR: 0.74, 95% CI: 0.62-0.87, versus *I^2^ =* 0%, HR: 0.59, 95% CI: 0.46-0.75, *P=*0.14).

**Figure 4:**
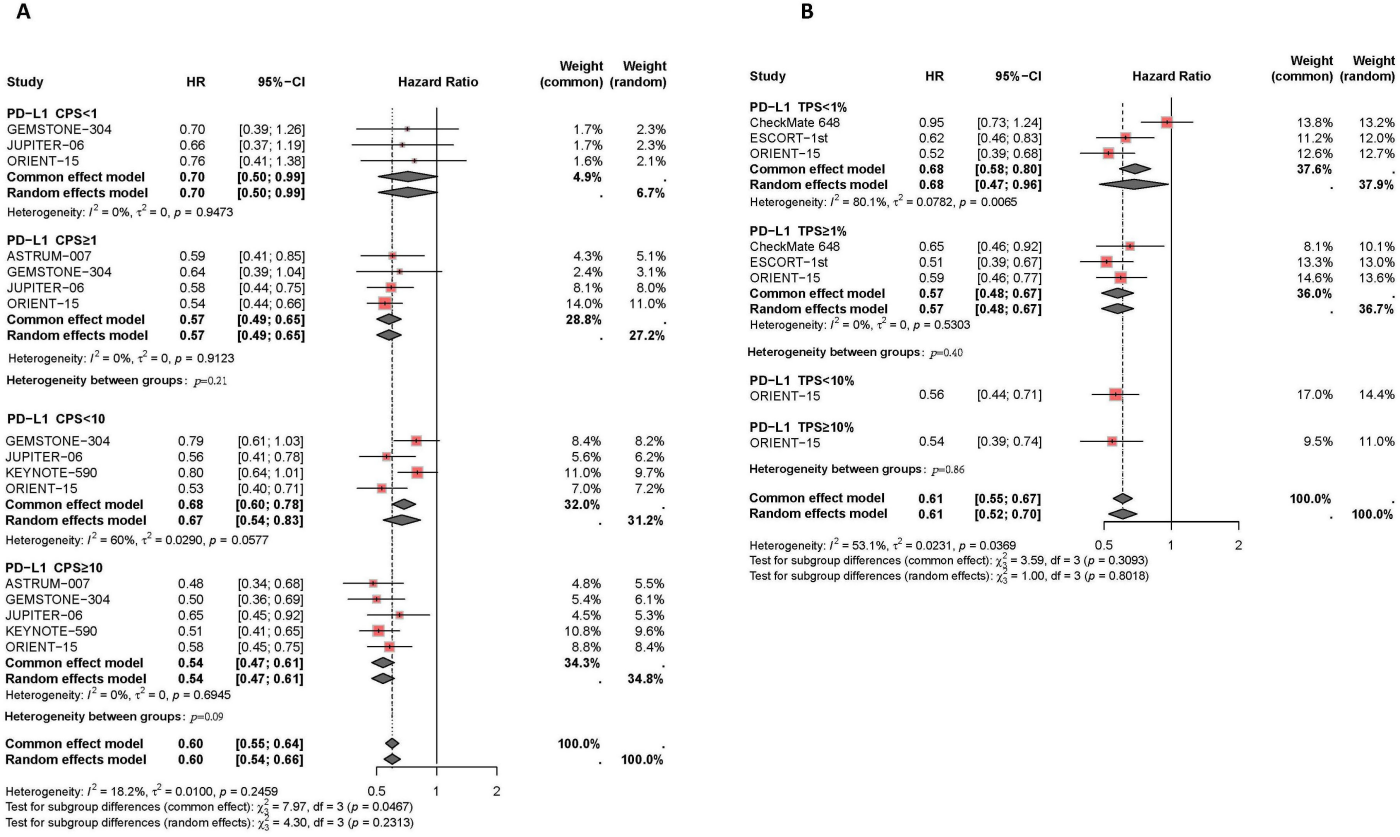
Forest plot of subgroup analysis comparing the progression-free survival HR in patients who received PD-1/PD-L1 inhibitor-based therapy versus chemotherapy based on different PDL1 expression levels of CPS **(A)** and TPS **(B)**.

Moreover, patients with higher PD-L1 expression levels experienced more effective disease control and better PFS benefits with PD-1/PD-L1 inhibitor therapy compared to those with lower PD-L1 expression levels, as shown in **Figure 4**. Subgroup analysis based on CPS thresholds revealed significant differences between groups (*P*=0.05). In patients with CPS<1, PD-1/PD-L1 inhibitor therapy did not significantly delay disease progression compared to chemotherapy alone (*I^2^ =* 0%, HR: 0.70, 95% CI: 0.50-0.99). However, in patients with CPS≥1, the combination of PD-1/PD-L1 inhibitors and chemotherapy effectively controlled disease progression compared to the control group (*I^2^ =* 0%, HR: 0.57, 95% CI: 0.49-0.65). In patients with CPS≥10, the combination therapy showed a greater reduction in disease progression compared to those with CPS<10 (*I^2^ =* 0%, HR: 0.54, 95% CI: 0.47-0.61, versus *I^2^ =* 60%, HR: 0.67, 95% CI: 0.50-0.78, *P*=0.09). Subgroup analysis based on TPS thresholds did not show significant differences between groups (*P*=0.31). Compared to patients with TPS<1% (*I^2^ =* 80%, HR: 0.68, 95% CI: 0.47-0.96), those with TPS≥1% experienced a slight delay in disease progression with the combination therapy compared to the control group (*I^2^ =* 0%, HR: 0.57, 95% CI: 0.48-0.67). Additionally, compared to patients with TPS <10% (HR: 0.56, 95% CI: 0.44-0.71), those with TPS ≥10% experienced better disease control with combined PD-1/PD-L1 inhibitors and chemotherapy (HR: 0.54, 95% CI: 0.39-0.74).

### 3.4 Subgroup analysis

**Figure 5:**
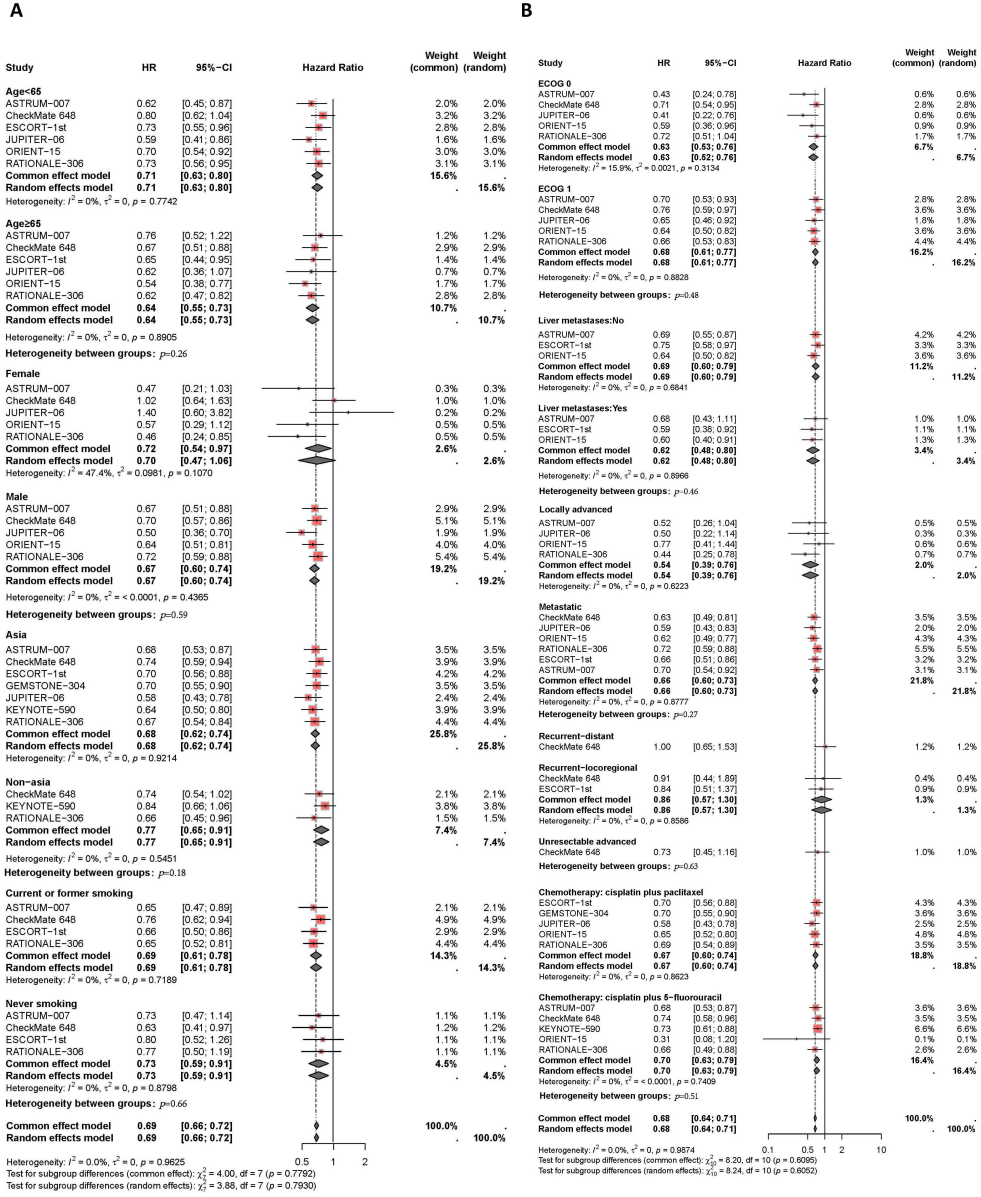
Forest plot of subgroup analyses by demographic characteristics **(A)** and clinical status **(B)** comparing overall survival in patients who received PD-1/PD-L1 inhibitor-based therapy versus chemotherapy.

**Figure 6:**
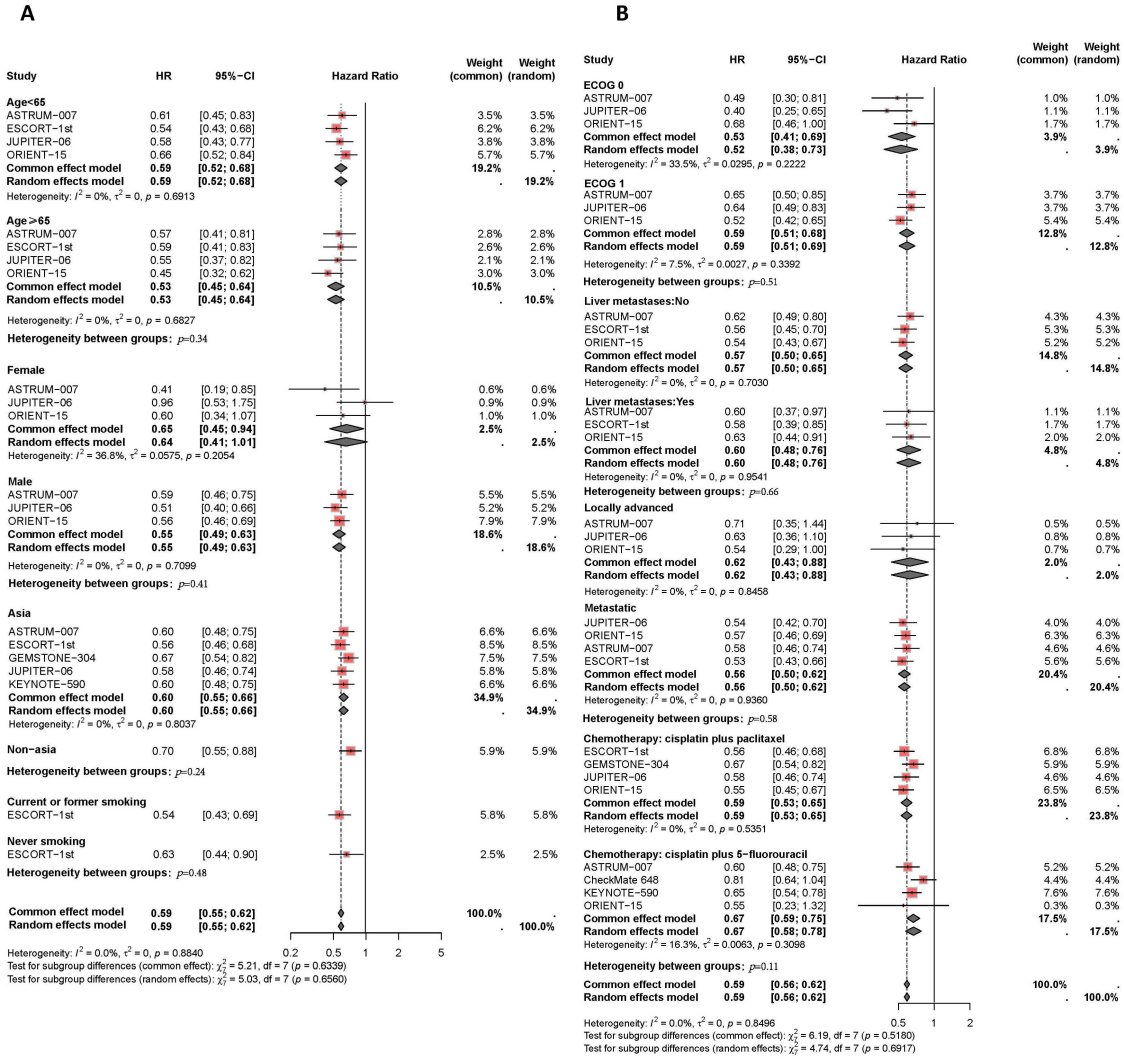
Forest plot of subgroup analyses by demographic characteristics **(A)** and clinical status **(B)** comparing progression-free survival in patients who received PD-1/PD-L1 inhibitor-based therapy versus chemotherapy.

Results of the subgroup analysis are presented in **Figures 5**, **6**. The OS and PFS HRs for patients receiving combination therapy were analyzed based on demographic and clinical characteristics, including age, gender, race, smoking status, Eastern Cooperative Oncology Group (ECOG) performance status, liver metastasis, recurrence status, and first-line chemotherapy regimen. **Figure 5** depicts survival improvements across different subgroups treated with PD-1/PD-L1 inhibitors. Male patients showed a slight trend toward better OS improvement with combination therapy compared to female patients (*I^2^ =* 0%, HR: 0.67, 95% CI: 0.60-0.74, versus *I^2^ =* 47%, HR: 0.72, 95% CI: 0.54-0.97, *P*=0.59). Similarly, Asian patients tended to show better OS outcomes from PD-L1 inhibitors plus chemotherapy compared to non-Asian patients (*I^2^ =* 0%, HR: 0.68, 95% CI: 0.62-0.74, versus *I^2^ =* 0%, HR: 0.77, 95% CI: 0.65-0.91, *P*=0.18). Smokers showed a trend toward greater OS benefit from combination therapy compared to non-smokers (*I^2^ =* 0%, HR: 0.69, 95% CI: 0.61-0.78, versus *I^2^ =* 0%, HR: 0.73, 95% CI: 0.59-0.91, *P*=0.66). Patients with liver metastases tended to have a greater reduction in mortality risk with immunotherapy compared to those without liver metastases (*I^2^ =* 0%, HR: 0.62, 95% CI: 0.48-0.80, versus *I^2^ =* 0%, HR: 0.69, 95% CI: 0.60-0.79, *P*=0.46). Regarding chemotherapy regimens, patients receiving PD-1/PD-L1 inhibitors combined with TP demonstrated similar OS benefits to those receiving PD-1/PD-L1 inhibitors plus FP (*I^2^ =* 0%, HR: 0.67, 95% CI: 0.50-0.74, versus *I^2^ =* 0%, HR: 0.70, 95% CI: 0.63-0.79, *P*=0.51). Additionally, subgroup analyses revealed comparable OS benefits among patients with different ECOG scores, progression statuses, recurrence statuses, and among those aged ≥65 years versus <65 years.

Finally, the PFS benefits of immunotherapy across different subgroups are presented in **Figure 6**. Compared to female patients (*I^2^ =* 37%, HR: 0.65, 95% CI: 0.45-0.94), male patients (*I^2^ =* 0%, HR: 0.55, 95% CI: 0.49-0.63) experienced a slight improvement in PFS with combination therapy (*P*=0.41). Asian patients receiving PD-L1 inhibitor plus chemotherapy had similar disease control compared to non-Asian patients (*I^2^ =* 0%, HR: 0.60, 95% CI: 0.55-0.56, versus HR: 0.70, 95% CI: 0.55-0.88, *P*=0.24). The efficacy of immunotherapy in patients with liver metastases was comparable to that in patients without liver metastases (*I^2^ =* 0%, HR: 0.60, 95% CI: 0.48-0.76, versus *I^2^ =* 0%, HR: 0.57, 95% CI: 0.50-0.65, *P*=0.66). Among different chemotherapy regimens, patients receiving PD-1/PD-L1 inhibitors combined with TP had slightly better disease control compared to those receiving PD-1/PD-L1 inhibitors plus FP (*I^2^ =* 0%, HR: 0.59, 95% CI: 0.53-0.65, versus *I^2^ =* 16%, HR: 0.67, 95% CI: 0.59-0.75, *P*=0.11).

### 3.5 Adverse events

**Figure 7:**
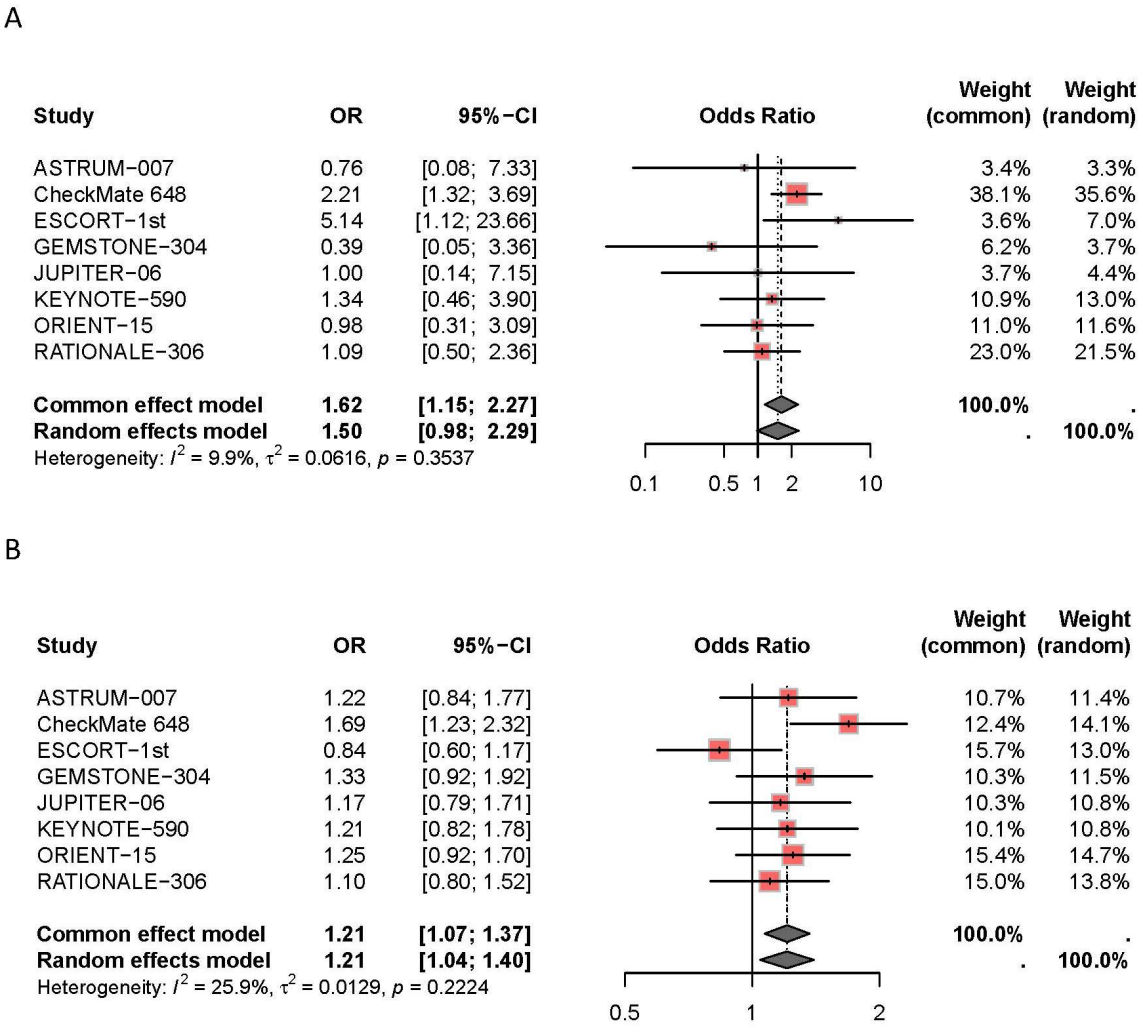
Subgroup analyses of AEs **(A)** and AEs ≥ grade 3 **(B)**.

The meta-analysis results concerning adverse events are presented in **Figure 7**. The results indicate that combination therapy with PD-1/PD-L1 inhibitors and chemotherapy was associated with an increased incidence of severe adverse events (≥grade 3) compared to chemotherapy alone (*I^2^ =* 26%, HR: 1.21, 95% CI: 1.07-1.37), and a higher overall adverse event rate (*I^2^ =* 10%, HR: 1.62, 95% CI: 1.15-2.27). Specifically, the CheckMate 648 trial demonstrated a significant increase in the incidence of severe adverse events with combination therapy (HR: 1.69, 95% CI: 1.23-2.32). Additionally, the ESCORT-1st trial reported a notable increase in the overall adverse event rate with combination therapy (HR: 5.14, 95% CI: 1.12-23.66).

### 3.6 Sensitivity analysis

**Figure 8:**
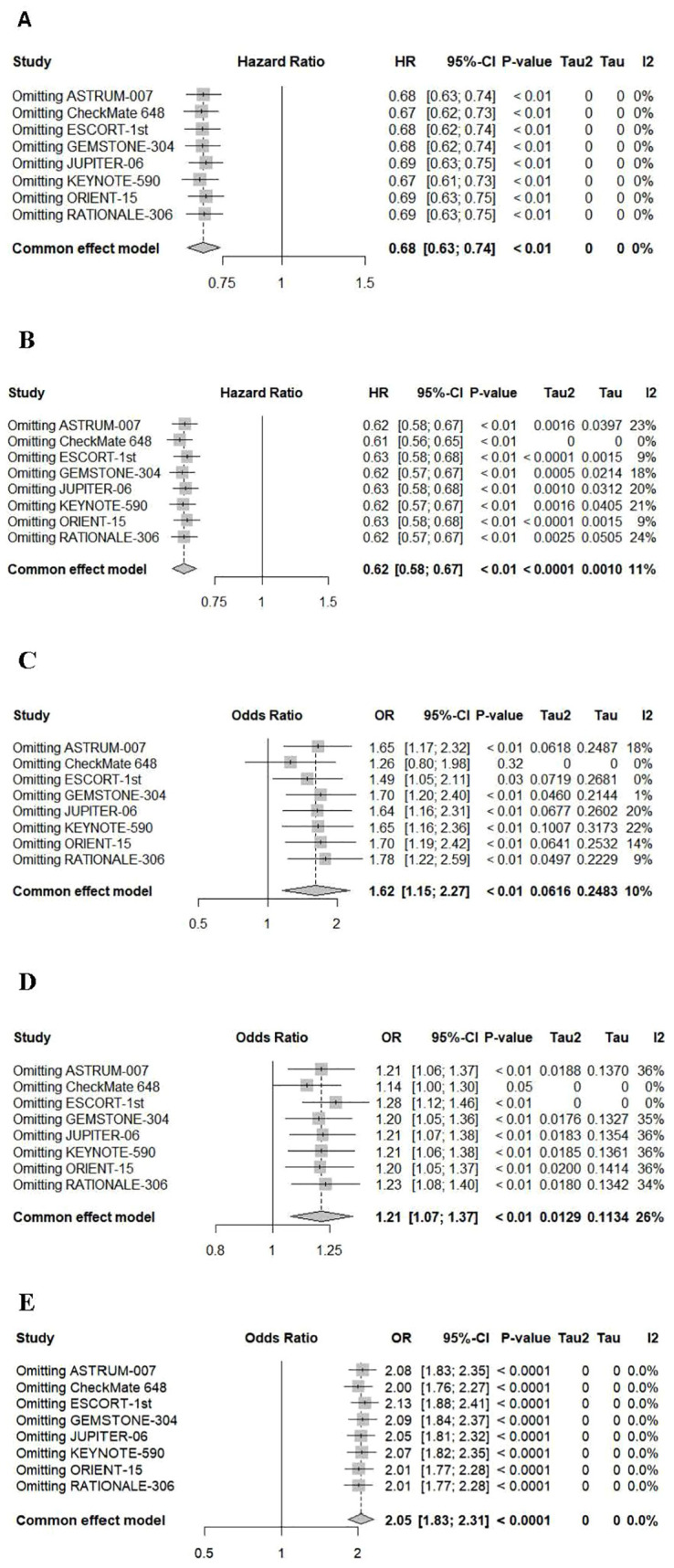
Sensitivity analyses of hazard ratios of overall survival **(A)**, progression-free survival **(B)** and adverse events **(C)**, severe adverse events (≥grade 3) **(D)** and ORR **(E)**.

A sensitivity analysis was conducted using a one-by-one exclusion method to evaluate the robustness of the research results (**Figure 8**). The summary HRs for OS, PFS, and adverse events ≥grade 3, as well as the summary OR of ORR, remained largely unchanged. However, the results for adverse events were influenced by the CheckMate 648 trial, and the overall effect size fluctuated significantly after excluding this study.

## 4 Discussion

ESCC, a highly invasive and aggressive malignancy of the digestive system, is associated with a poor prognosis. While PD-1/PD-L1 inhibitors have shown promise as a novel immunotherapeutic approach, their combined efficacy with different chemotherapy regimens remains unclear. The main objective of our study was to systematically evaluate the efficacy and potential influencing factors of PD-1/PD-L1 inhibitors in the treatment of ESCC by comprehensively analyzing data from multiple phase III RCTs. The findings of our study suggest that CPS may serve as a superior predictor of patient response to PD-1/PD-L1 inhibitors compared to TPS and could function as a more reliable biomarker for assessing PD-L1 expression levels. Additionally, subgroup analyses indicated that male patients, Asian patients, and those with liver metastases might derive greater OS benefits and better disease control from combination therapy with PD-1/PD-L1 inhibitors and chemotherapy.

Recently, the combination of ICIs and chemotherapy has emerged as a promising strategy for treating esophageal cancer, potentially offering synergistic effects that enhance treatment outcomes (28, 29). The current meta-analysis found that PD-1/PD-L1 inhibitors combined with chemotherapy significantly improved therapeutic efficacy (30, 31). However, the response to combination therapy varied among patients with different PD-L1 expression levels. Previous meta-analyses in gastric cancer have shown CPS was superior to TPS, with CPS=1, CPS=5, and CPS=10 serving as potential thresholds for improved OS in gastric cancer patients receiving ICIs (19). By comparing the improved effectiveness of inhibitors across different PD-L1 expression levels using TPS and CPS, it was found that the PD-L1 CPS threshold seemed to be a more reliable predictive factor for reducing mortality when using PD-1/PD-L1 inhibitors than the TPS threshold. As a PD-L1 expression score system, CPS may be more meaningful for predicting patient prognosis. However, there are limited studies on the predictive value of CPS and TPS for esophageal cancer combination therapies. The subgroup analysis of the current meta-analysis, based on different PD-L1 expression levels and assessment methods, demonstrated that compared with TPS, CPS as a PD-L1 expression evaluation method had more significant inter-group differences. The HR for OS or PFS in patients with PD-L1 CPS≥1 was lower than that in patients with PD-L1 CPS<1, and the difference was statistically significant in patients with PD-L1 CPS ≥10 versus <10. Therefore, CPS can better predict patients’ response to PD-1/PD-L1 inhibitors. Patients with positive PD-L1 expression benefit more in terms of survival than those with negative PD-L1 expression, and CPS ≥10 as the key threshold can more significantly distinguish and predict patients’ efficacy. This is consistent with previous expert opinions (30). During the FDA ODAC (Oncologic Drugs Advisory Committee) meeting on September 26, 2024, the risks and benefits of the treatment with anti-PD-1 antibodies for the first-line treatment of patients with unresectable or metastatic ESCC with PD-L1 expression <1 were discussed. Most panelists voted that patients with PD-L1 expression <1 are unlikely to benefit from first-line treatment with PD-1 inhibitors, given the associated risks (32). The result of the FDA ODAC meeting further suggests that advanced ESCC patients with positive PD-L1 expression may benefit more from immunotherapy.

Recent studies have suggested that the response of ESCC patients to PD-1/PD-L1 inhibitors may be influenced by various factors, including age, gender, and ethnicity (33–35). Subgroup analysis in the current study found that male patients benefit more from immunotherapy compared to female patients. Traditionally, it was believed that female patients with ESCC had better chemotherapy outcomes than males. However, the current analysis found that males benefit more from combined immunotherapy, possibly because males were more predominantly affected by esophageal cancer and tend to have less benefit from chemotherapy alone, making them more likely to gain from combined treatments (36, 37). Over 60% of esophageal cancer patients globally live in East Asia, where squamous cell carcinoma is the predominant histological type (38, 39). Gao et al. (40) compared pathological responses to neoadjuvant chemoradiotherapy between Eastern and Western countries and found poorer responses among ESCC patients in Eastern countries. Additionally, some studies suggested that differences in treatment response between Caucasian and Asian patients are related to variations in gene polymorphisms affecting drug metabolism and DNA repair (41, 42). ESCC shows significant racial differences in the efficacy response to chemotherapy, with East Asian patients often having poorer responses. In terms of immunotherapy, experimental results indicated that the OS benefit for Chinese subgroups is three times that of the overall study population (33). This aligns with our findings, where the subgroup analysis shows better efficacy in Asian patients compared to non-Asians. This may be due to distinct immune system characteristics in Asian patients. Further studies are needed to explore the unique mechanisms of response in Asian patients undergoing combined immunotherapy and chemotherapy, focusing on genetic, immune features, and tumor microenvironment factors.

The selection of an appropriate chemotherapy regimen in combination therapy with ICIs and chemotherapy could maximize treatment efficacy and patient survival rates (43). Different chemotherapy regimens have varying implications in combination treatments. Traditionally, the TP regimen was considered superior to the FP regimen for treating ESCC, but previous studies have shown inconsistent findings (44). Some retrospective studies indicated that TP and FP regimens show similar efficacy with no statistical differences between the groups (44). However, Meta-analyses (13, 45) of multiple clinical trials evaluating the efficacy and safety of TP and FP regimens in ESCC treatment revealed that PD-1+TP significantly improves PFS and OS compared to PD-1+FP, suggesting that PD-1+TP may be the best first-line treatment option. Subgroup analysis in our study shows no significant difference between PD-1/PD-L1 inhibitors combined with TP and FP regimens, but patients receiving ICIs+TP had lower HRs for OS and PFS compared to those receiving ICIs+FP. The short-term efficacy of TP regimen, as measured by PFS, may be slightly better than FP, while the difference in long-term efficacy, OS, between the two regimens was very small. This discrepancy may be due to variations in patients’ pathological features and clinical conditions, leading to differences in regimen efficacy. Paclitaxel could enhance immune cell activation and synergize with immune therapy by modulating the immune microenvironment, promoting cancer cell apoptosis (46, 47). In contrast, fluorouracil (5-FU), an antimetabolite, inhibits DNA and RNA synthesis, while cisplatin induces DNA damage, together providing a comprehensive anti-cancer effect. The impact of chemotherapy regimens may exhibit heterogeneity among patients, so treatment adjustments should consider specific factors such as age, tumor type, stage, and overall health status.

Our study conducted a meta-analysis of multiple phase III RCTs to provide a comprehensive evaluation of PD-1/PD-L1 inhibitors in ESCC treatment. We found that combination therapy has shown significant benefits and evaluated the combined effects of different chemotherapy regimens (TP and FP) with immunotherapy. The differences in treatment efficacy between different chemotherapy regimens were not significant, which may affect treatment decision-making. In patients with PD-L1 CPS ≥1, the combination of PD-1/PD-L1 inhibitors and chemotherapy effectively controlled disease progression and reduced the risk of death compared to the control group. Additionally, the PD-L1 CPS system may be more sensitive in predicting treatment outcomes than the TPS system, and our study identified PD-L1 CPS expression level as a predictor of survival benefit from PD-1/PD-L1 inhibitor therapy, providing valuable insights for future clinical application. Finally, the study suggested that male, Asian, and liver metastasis patients may benefit more from combination therapy, though these differences were not significant. Further in-depth molecular and immune mechanism research is needed to explore the immune microenvironment, cytokine profile, and immune-related gene expression in patients of different genders, races, and disease progression.

While this study provides important insights, several limitations should be acknowledged. First, since a higher proportion of enrolled participants were of Asian descent (consistent with regional epidemiology), this geographic concentration may limit the generalizability of the findings to Caucasian and African populations. Second, substantial methodological heterogeneity was observed in PD-L1 assessment, stemming from divergent immunohistochemical platforms (Dako22C3 versus Ventana SP263) and scoring systems (CPS versus TPS), thereby impeding comparative analyses. Additionally, the follow-up duration for survival outcomes was relatively short, with long-term survival data not being reported, limiting the ability to assess durable therapeutic benefits. In terms of safety data, adverse events were pooled without stratification by severity, and rare but serious adverse events may be underrepresented due to trial exclusion criteria. Furthermore, the inclusion of only phase 3 trials may omit negative or smaller studies, potentially introducing publication bias. Lastly, beyond PD-L1, other predictive biomarkers such as tumor mutational burden (TMB) and microsatellite instability (MSI) were not analyzed, limiting insights into precision treatment strategies.

In summary, the current study found that the combination of ICIs and chemotherapy showed significant therapeutic benefits in the treatment of ESCC. There was no significant difference in the therapeutic effect between the combination of immunotherapy with the TP regimen and the FP regimen in ESCC, although the TP regimen had slightly better short-term efficacy than the FP regimen. CPS may be more effective than TPS in predicting the efficacy of PD-1/PD-L1 inhibitors. Patients with CPS >1 showed a more significant therapeutic response, with CPS of 10 being the key threshold for predicting patient response. Male, Asian, and liver metastasis patients may derive slightly better survival outcomes from the combination therapy. Further research is needed to investigate the reliability and thresholds of CPS and TPS in larger clinical trials, exploring relevant influencing factors to confirm their reliability and consistency across different populations and treatment scenarios.

## Data Availability

The original contributions presented in the study are included in the article. Further inquiries can be directed to the corresponding authors.

## References

[1] Bray F Laversanne M Sung H Ferlay J Siegel RL Soerjomataram I . Global cancer statistics 2022: GLOBOCAN estimates of incidence and mortality worldwide for 36 cancers in 185 countries. CA Cancer J Clin. (2024) 74:229–63. doi: 10.3322/caac.21834 38572751

[2] Siegel RL Miller KD Jemal A . Cancer statistics, 2020. CA Cancer J Clin. (2020) 70:7–30. doi: 10.3322/caac.21590 31912902

[3] Yang H Wang F Hallemeier CL Lerut T Fu J . Oesophageal cancer. Lancet. (2024) 404:1991–2005. doi: 10.1016/s0140-6736(24)02226-8 39550174

[4] Abnet CC Arnold M Wei WQ . Epidemiology of esophageal squamous cell carcinoma. Gastroenterology. (2018) 154:360–73. doi: 10.1053/j.gastro.2017.08.023 PMC5836473 28823862

[5] Kamangar F Chow WH Abnet CC Dawsey SM . Environmental causes of esophageal cancer. Gastroenterol Clin North Am. (2009) 38:27–57. doi: 10.1016/j.gtc.2009.01.004 19327566 PMC2685172

[6] Islami F Kamangar F Aghcheli K Fahimi S Semnani S Taghavi N . Epidemiologic features of upper gastrointestinal tract cancers in Northeastern Iran. Br J Cancer. (2004) 90:1402–6. doi: 10.1038/sj.bjc.6601737 PMC2409685 15054463

[7] Wang SM Abnet CC Qiao YL . What have we learned from Linxian esophageal cancer etiological studies? Thorac Cancer. (2019) 10:1036–42. doi: 10.1111/1759-7714.13058 PMC6500974 30925028

[8] Puhr HC Prager GW Ilhan-Mutlu A . How we treat esophageal squamous cell carcinoma. ESMO Open. (2023) 8:100789. doi: 10.1016/j.esmoop.2023.100789 36791637 PMC9958251

[9] Zeng H Zhang F Sun Y Li S Zhang W . Treatment options for neoadjuvant strategies of esophageal squamous cell carcinoma (Review). Mol Clin Oncol. (2024) 20:4. doi: 10.3892/mco.2023.2702 38223404 PMC10784769

[10] Lu Z Wang J Shu Y Liu L Kong L Yang L . Sintilimab versus placebo in combination with chemotherapy as first line treatment for locally advanced or metastatic oesophageal squamous cell carcinoma (ORIENT-15): multicentre, randomised, double blind, phase 3 trial. BMJ. (2022) 377:e068714. doi: 10.1136/bmj-2021-068714 35440464 PMC9016493

[11] Joyce JA Fearon DT . T cell exclusion, immune privilege, and the tumor microenvironment. Science. (2015) 348:74–80. doi: 10.1126/science.aaa6204 25838376

[12] He S Xu J Liu X Zhen Y . Advances and challenges in the treatment of esophageal cancer. Acta Pharm Sin B. (2021) 11:3379–92. doi: 10.1016/j.apsb.2021.03.008 PMC8642427 34900524

[13] Li Y Ji Y Shen L Huang T Deng B Guo H . Clinical efficacy of combination therapy of an immune checkpoint inhibitor with taxane plus platinum versus an immune checkpoint inhibitor with fluorouracil plus platinum in the first-line treatment of patients with locally advanced, metastatic, or recurrent esophageal squamous cell carcinoma. Front Oncol. (2022) 12:1015302. doi: 10.3389/fonc.2022.1015302 36605427 PMC9808083

[14] Sun JM Shen L Shah MA Enzinger P Adenis A Doi T . Pembrolizumab plus chemotherapy versus chemotherapy alone for first-line treatment of advanced oesophageal cancer (KEYNOTE-590): a randomised, placebo-controlled, phase 3 study. Lancet. (2021) 398:759–71. doi: 10.1016/s0140-6736(21)01234-4 34454674

[15] Wang ZX Cui C Yao J Zhang Y Li M Feng J . Toripalimab plus chemotherapy in treatment-naïve, advanced esophageal squamous cell carcinoma (JUPITER-06): A multi-center phase 3 trial. Cancer Cell. (2022) 40:277–288 e3. doi: 10.1016/j.ccell.2022.02.007 35245446

[16] Geng Y Zhang Q Feng S Li C Wang L Zhao X . Safety and Efficacy of PD-1/PD-L1 inhibitors combined with radiotherapy in patients with non-small-cell lung cancer: a systematic review and meta-analysis. Cancer Med. (2021) 10:1222–39. doi: 10.1002/cam4.v10.4 PMC7926021 33465302

[17] Fuchs CS Özgüroğlu M Bang YJ Di Bartolomeo M Mandala M Ryu MH . Pembrolizumab versus paclitaxel for previously treated PD-L1-positive advanced gastric or gastroesophageal junction cancer: 2-year update of the randomized phase 3 KEYNOTE-061 trial. Gastric Cancer. (2022) 25:197–206. doi: 10.1007/s10120-021-01227-z 34468869 PMC8732941

[18] Boku N Satoh T Ryu MH Chao Y Kato K Chung HC . Nivolumab in previously treated advanced gastric cancer (ATTRACTION-2): 3-year update and outcome of treatment beyond progression with nivolumab. Gastric Cancer. (2021) 24:946–58. doi: 10.1007/s10120-021-01173-w PMC8205916 33743112

[19] Noori M Fayyaz F Zali MR Bashash D . Predictive value of PD-L1 expression in response to immune checkpoint inhibitors for gastric cancer treatment: a systematic review and meta-analysis. Expert Rev Anticancer Ther. (2023) 23:1029–39. doi: 10.1080/14737140.2023.2238896 37466449

[20] Metges JP Kato K Sun JM . First-line pembrolizumab plus chemotherapy versus chemotherapy in advanced esophageal cancer: Longer-term efficacy, safety, and quality-of-life results from the phase 3 KEYNOTE-590 study. Conference Abstract. J Clin Oncol. (2022) 40(4_suppl):241. doi: 10.1200/JCO.2022.40.4-suppl.241

[21] Li J Chen Z Bai Y Liu B Li Q Zhou J . GEMSTONE-304: a phase 3 study of sugemalimab plus chemotherapy versus chemotherapy as first-line treatment of patients with unresectable locally advanced, recurrent or metastatic esophageal squamous cell carcinoma (ESCC). Conf proceeding Ann Oncol. (2023) 34:S181–2. doi: 10.1016/j.annonc.2023.04.019

[22] Kojima T Hara H Tsuji A Yasui H Muro K Satoh T . First-line pembrolizumab + chemotherapy in Japanese patients with advanced/metastatic esophageal cancer from KEYNOTE-590. Esophagus. (2022) 19:683–92. doi: 10.1007/s10388-022-00920-x PMC9436840 35668304

[23] Kato K Doki Y Ogata T Motoyama S Kawakami H Ueno M . First-line nivolumab plus ipilimumab or chemotherapy versus chemotherapy alone in advanced esophageal squamous cell carcinoma: a Japanese subgroup analysis of open-label, phase 3 trial (CheckMate 648/ONO-4538-50). Esophagus. (2023) 20:291–301. doi: 10.1007/s10388-022-00970-1 36401133 PMC10024660

[24] Xu J Kato K Raymond E Hubner RA Shu Y Pan Y . Tislelizumab plus chemotherapy versus placebo plus chemotherapy as first-line treatment for advanced or metastatic oesophageal squamous cell carcinoma (RATIONALE-306): a global, randomised, placebo-controlled, phase 3 study. Lancet Oncol. (2023) 24:483–95. doi: 10.1016/s1470-2045(23)00108-0 37080222

[25] Doki Y Ajani JA Kato K Xu J Wyrwicz L Motoyama S . Nivolumab combination therapy in advanced esophageal squamous-cell carcinoma. New Engl J Med. (2022) 386:449–62. doi: 10.1056/NEJMoa2111380 35108470

[26] Song Y Zhang B Xin D Kou X Tan Z Zhang S . First-line serplulimab or placebo plus chemotherapy in PD-L1-positive esophageal squamous cell carcinoma: a randomized, double-blind phase 3 trial. Nat Med. (2023) 29:473–82. doi: 10.1038/s41591-022-02179-2 PMC9941045 36732627

[27] Luo H Lu J Bai Y Mao T Wang J Fan Q . Effect of camrelizumab vs placebo added to chemotherapy on survival and progression-free survival in patients with advanced or metastatic esophageal squamous cell carcinoma: the ESCORT-1st randomized clinical trial. Jama. (2021) 326:916–25. doi: 10.1001/jama.2021.12836 PMC8441593 34519801

[28] Wang R Liu S Chen B Xi M . Recent advances in combination of immunotherapy and chemoradiotherapy for locally advanced esophageal squamous cell carcinoma. Cancers (Basel). (2022) 14(20):5168. doi: 10.3390/cancers14205168 36291954 PMC9599968

[29] Salas-Benito D Pérez-Gracia JL Ponz-Sarvisé M Rodriguez-Ruiz ME Martínez-Forero I Castañón E . Paradigms on immunotherapy combinations with chemotherapy. Cancer Discovery. (2021) 11:1353–67. doi: 10.1158/2159-8290.Cd-20-1312 33712487

[30] Lu Y Wang W Wang F . Clinical benefits of PD-1 inhibitors in specific subgroups of patients with advanced esophageal squamous cell carcinoma: a systematic review and meta-analysis of phase 3 randomized clinical trials. Front Immunol. (2023) 14:1171671. doi: 10.3389/fimmu.2023.1171671 37205107 PMC10185849

[31] Lu Y Xu M Guan L Yang Y Chen Y Yang Y . PD-1 inhibitor plus chemotherapy versus chemotherapy as first-line treatment for advanced esophageal cancer: A systematic review and meta-analysis. J Immunother. (2022) 45:243–53. doi: 10.1097/CJI.0000000000000420 PMC9087869 35467579

[32] U.S. Food and Drug Administration . Meeting of the oncologic drugs advisory committee meeting announcement (2024). Available online at: https://www.fda.gov/media/182143/download (Accessed April 12, 2024).

[33] Cao Y Qin S Luo S Li Z Cheng Y Fan Y . Pembrolizumab versus chemotherapy for patients with esophageal squamous cell carcinoma enrolled in the randomized KEYNOTE-181 trial in Asia. ESMO Open. (2022) 7:100341. doi: 10.1016/j.esmoop.2021.100341 34973513 PMC8764510

[34] Kauppila JH Wahlin K Lagergren P Lagergren J . Sex differences in the prognosis after surgery for esophageal squamous cell carcinoma and adenocarcinoma. Int J Cancer. (2019) 144:1284–91. doi: 10.1002/ijc.31840 30168595

[35] Haupt S Caramia F Klein SL Rubin JB Haupt Y . Sex disparities matter in cancer development and therapy. Nat Rev Cancer. (2021) 21:393–407. doi: 10.1038/s41568-021-00348-y 33879867 PMC8284191

[36] Athauda A Nankivell M Langley RE Alderson D Allum W Grabsch HI . Impact of sex and age on chemotherapy efficacy, toxicity and survival in localised oesophagogastric cancer: A pooled analysis of 3265 individual patient data from four large randomised trials (OE02, OE05, MAGIC and ST03). Eur J Cancer. (2020) 137:45–56. doi: 10.1016/j.ejca.2020.06.005 32745964

[37] Sukocheva OA Li B Due SL Hussey DJ Watson DI . Androgens and esophageal cancer: What do we know? World J Gastroenterol. (2015) 21:6146–56. doi: 10.3748/wjg.v21.i20.6146 PMC4445092 26034350

[38] Eyck BM Gao X Yang Y van der Wilk BJ Wong I Wijnhoven BPL . Pathological response to neoadjuvant chemoradiotherapy for oesophageal squamous cell carcinoma: multicentre East Asian and Dutch database comparison. Br J Surg. (2022) 109:1312–8. doi: 10.1093/bjs/znac314 PMC10364703 36036665

[39] Arnold M Ferlay J van Berge Henegouwen MI Soerjomataram I . Global burden of oesophageal and gastric cancer by histology and subsite in 2018. Gut. (2020) 69:1564–71. doi: 10.1136/gutjnl-2020-321600 32606208

[40] Gao X Overtoom HCG Eyck BM Huang SH Nieboer D van der Sluis PC . Pathological response to neoadjuvant chemoradiotherapy for oesophageal squamous cell carcinoma in Eastern versus Western countries: meta-analysis. Br J Surg. (2024) 111(5):znae083. doi: 10.1093/bjs/znae083 38721902

[41] Millward MJ Boyer MJ Lehnert M Clarke S Rischin D Goh BC . Docetaxel and carboplatin is an active regimen in advanced non-small-cell lung cancer: a phase II study in Caucasian and Asian patients. Ann Oncol Mar. (2003) 14:449–54. doi: 10.1093/annonc/mdg118 12598352

[42] Gandara DR Kawaguchi T Crowley J Moon J Furuse K Kawahara M . Japanese-US common-arm analysis of paclitaxel plus carboplatin in advanced non-small-cell lung cancer: a model for assessing population-related pharmacogenomics. J Clin Oncol. (2009) 27:3540–6. doi: 10.1200/JCO.2008.20.8793 PMC2717760 19470925

[43] Noguchi T Moriyama H Wada S Takeno S Wakisaka M Mori H . Resection surgery with neoadjuvant chemoradiotherapy improves outcomes of patients with T4 esophageal carcinoma. Dis Esophagus. (2003) 16:94–8. doi: 10.1046/j.1442-2050.2003.00304.x 12823205

[44] Liu Y Ren Z Yuan L Xu S Yao Z Qiao L . Paclitaxel plus cisplatin vs. 5-fluorouracil plus cisplatin as first-line treatment for patients with advanced squamous cell esophageal cancer. Am J Cancer Res. (2016) 6(10):2345–50. PMC5088297 27822423

[45] Zhao J Zhang S Guo X Li C Yang B Qu X . PD-1 inhibitors combined with paclitaxel and cisplatin in first-line treatment of esophageal squamous cell carcinoma (ESCC): a network meta-analysis. BMC Cancer. (2023) 23:1221. doi: 10.1186/s12885-023-11715-3 38082441 PMC10714592

[46] Garnett CT Schlom J Hodge JW . Combination of docetaxel and recombinant vaccine enhances T-cell responses and antitumor activity: effects of docetaxel on immune enhancement. Clin Cancer Res. (2008) 14:3536–44. doi: 10.1158/1078-0432.CCR-07-4025 PMC2682419 18519787

[47] Galluzzi L Humeau J Buqué A Zitvogel L Kroemer G . Immunostimulation with chemotherapy in the era of immune checkpoint inhibitors. Nat Rev Clin Oncol. (2020) 17:725–41. doi: 10.1038/s41571-020-0413-z 32760014

