# Corrigendum: Efficacy and safety of PD-1/PD-L1 inhibitors as first-line treatment for esophageal squamous cell carcinoma: a systematic review and meta-analysis

**DOI:** 10.3389/fimmu.2025.1611591

**Published:** 2025-05-16

**Authors:** Wei Ren, Hanyu Zhang, Yixin Li, Wu Sun, Hexiang Peng, Huangda Guo, Tianjiao Hou, Mengying Wang, Zhendong Hu, Tao Wu, Baorui Liu

**Affiliations:** ^1^ The Comprehensive Cancer Center of Drum Tower Hospital, Medical School of Nanjing University and Clinical Cancer Institute of Nanjing University, Nanjing, China; ^2^ Department of Epidemiology and Biostatistics, School of Public Health, Peking University, Beijing, China; ^3^ Key Laboratory of Epidemiology of Major Diseases (Peking University), Ministry of Education, Beijing, China; ^4^ Department of Nutrition and Food Hygiene, School of Public Health, Peking University, Beijing, China; ^5^ Department of Esophageal Surgery, Drum Tower Hospital, Medical School of Nanjing University, Nanjing, China

**Keywords:** PD-1/PD-L1 inhibitor, esophageal squamous cell carcinoma, meta-analysis, immunotherapy, combined positive score

In the published article, there was an error in **Figure 3** and **Figure 4** as published. The trend line visualization in the original **Figure 3** was incomplete. The hierarchical annotation in the original **Figure 4** erroneously uses the symbol “=” instead of “≥” for stratification labels. The corrected **Figure 3** and **Figure 4** and its caption

**Figure 3:**
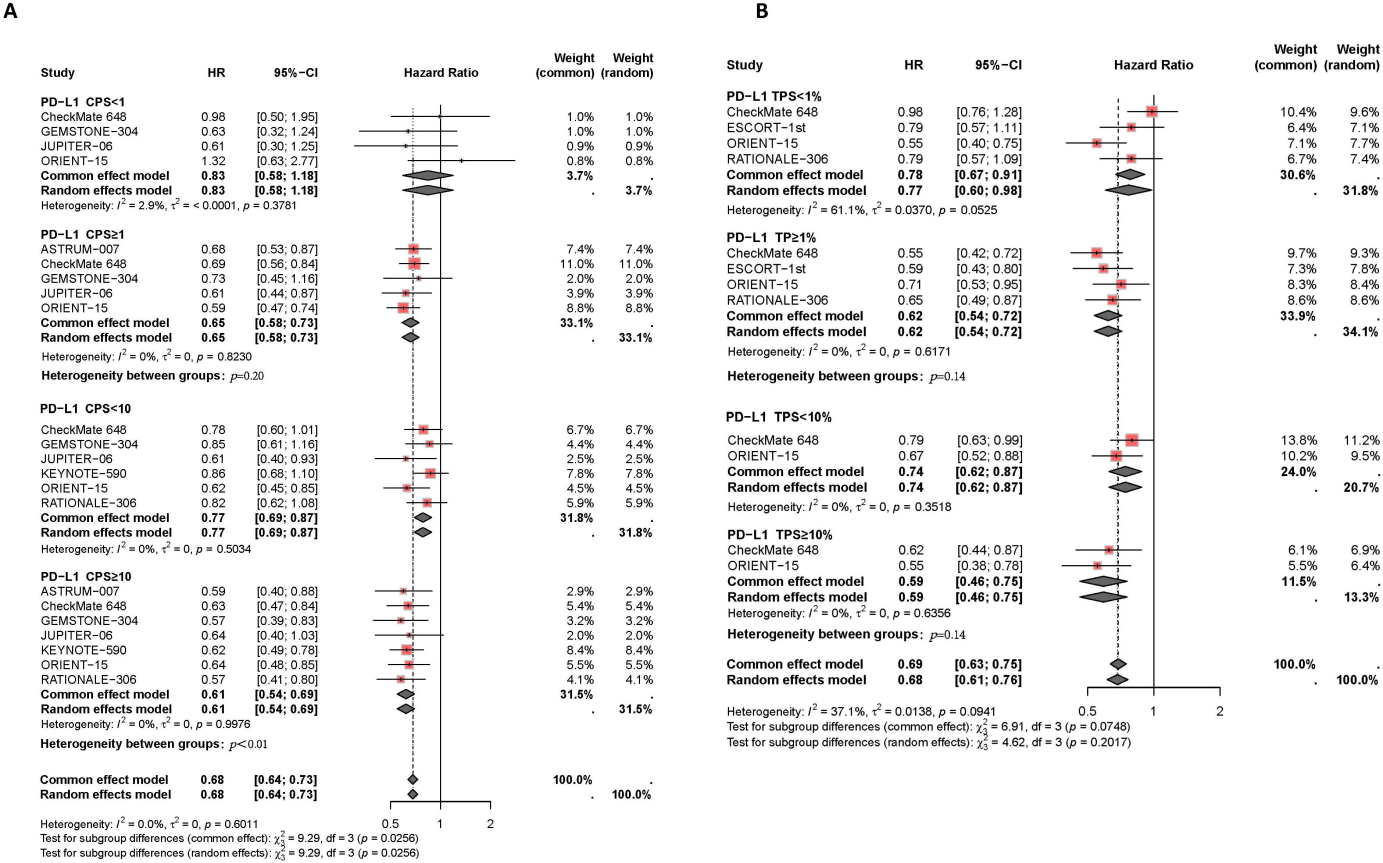
Forest plot of subgroup analysis comparing the overall survival HR in patients who received PD-1/PD-L1 inhibitor-based therapy versus chemotherapy based on different PDL1 expression levels of CPS **(A)** and TPS **(B)**.

**Figure 4:**
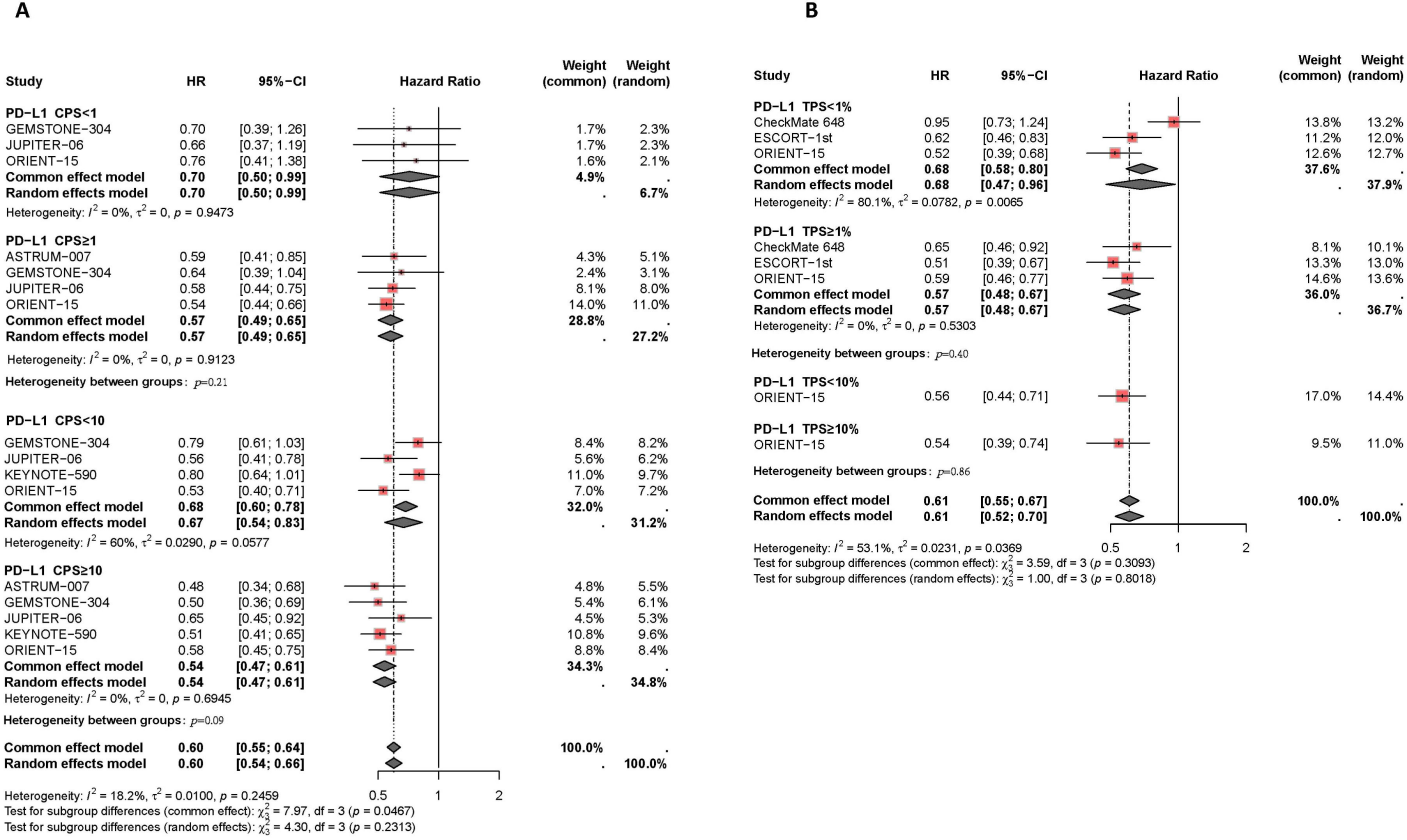
Forest plot of subgroup analysis comparing the progression-free survival HR in patients who received PD-1/PD-L1 inhibitor-based therapy versus chemotherapy based on different PDL1 expression levels of CPS **(A)** and TPS **(B)**.

“Figure 3 Forest plot of subgroup analysis comparing the overall survival HR in patients who received PD-1/PD-L1 inhibitor-based therapy versus chemotherapy based on different PDL1 expression levels of CPS (A) and TPS (B).”

“Figure 4 Forest plot of subgroup analysis comparing the progression-free survival HR in patients who received PD-1/PD-L1 inhibitor-based therapy versus chemotherapy based on different PDL1 expression levels of CPS (A) and TPS (B).” appear below.

The authors apologize for this error and state that this does not change the scientific conclusions of the article in any way. The original article has been updated.

